# Argon Ion Beam Current Dependence of Si-Si Surface Activated Bonding

**DOI:** 10.3390/ma15093115

**Published:** 2022-04-25

**Authors:** Song Yang, Ningkang Deng, Yongfeng Qu, Kang Wang, Yuan Yuan, Wenbo Hu, Shengli Wu, Hongxing Wang

**Affiliations:** 1 Key Laboratory for Physical Electronics and Devices of the Ministry of Education, School of Electronic Science and Engineering, Xi’an Jiaotong University, No. 28, Xianning West Road, Xi’an 710049, China; 18821623054@163.com (S.Y.); dnk0309@163.com (N.D.); yfq0924@163.com (Y.Q.); wangkang2005to2008@126.com (K.W.); slwu@mail.xjtu.edu.cn (S.W.); hxwangcn@mail.xjtu.edu.cn (H.W.); 2 Science and Technology on Low-Light-Level Night Vision Laboratory, Xi’an 710065, China; cater2046@163.com

**Keywords:** surface activated bonding, room-temperature Si-Si bonding, Ar ion beam irradiation, bonding interface

## Abstract

In order to optimize the process parameters of Si-Si wafer direct bonding at room temperature, Si-Si surface activated bonding (SAB) was performed, and the effect of the argon ion beam current for surface activation treatment on the Si-Si bonding quality was investigated. For the surface activation under the argon ion beam irradiation for 300 s, a smaller ion beam current (10~30 mA) helped to realize a lower percentage of area covered by voids and higher bonding strength. Especially with the surface activation under 30 mA, the bonded Si-Si specimen obtained the highest bonding quality, and its percentage of area covered by voids and bonding strength reached <0.2% and >7.62 MPa, respectively. The transmission electron microscopy analyses indicate that there exists an ultrathin amorphous Si interlayer at the Si-Si bonding interface induced by argon ion beam irradiation to Si wafer surfaces, and its thickness increases as the argon ion beam current rises. The investigation results can be used to optimize the SAB process and promote the applications of SAB in the field of semiconductor devices.

## 1. Introduction

As a kind of integration technology of heterogeneous materials or devices, wafer bonding can easily solve the issues induced by lattice and thermal mismatches between heterogeneous semiconductor materials in epitaxial growth technology [1,2,3]. As we know, the traditional bonding method needs a high-temperature process, which could lead to serious problems such as thermal stress caused by thermal mismatch [4] and broadening doping profile of the device [5,6]. In order to meet the requirements of microwave power electronic device fabrication [7,8,9,10,11] and MEMS packaging [12,13], atomic diffusion bonding and surface activated bonding (SAB) can be performed under the conditions of low temperature and interlayer-free (or super-thin interlayer), and have attracted extensive attention and been deeply studied.

Atomic diffusion bonding usually uses metal film from a few nanometers to tens of nanometers in thickness as an interlayer and takes advantage of metal atomic diffusion and recrystallization to bond two materials together [14,15]. This bonding technique has some advantages, such as a simple bonding machine, low temperature (even room temperature) process and relatively loose material surface roughness requirement, but it needs to apply a higher pressure force [16], which may cause the ruptures of materials or devices and introduces a metal interlayer, which could produce interference to the operation of the high-frequency device.

SAB utilizes fast atom (or ion) irradiation to activate the surfaces of two materials and generate the dangling bonds on these two surfaces, which leads to the formation of the chemical bonds between these two surfaces in contact with each other. The unique virtue of SAB is that it can realize the direct bonding of two materials without an interlayer under weak pressure force at room temperature, which is very conducive to the fabrication of heterogeneous semiconductor devices with extra-low thermal boundary resistance and considered to be a promising method to fabricate structurally novel devices with high frequency, high power and high integration. However, the atomic level smooth surface with roughness below 1 nm (even 0.5 nm) required by the SAB process increases its technical difficulty in comparison with atomic diffusion bonding. Nevertheless, in recent years SAB as an advanced bonding method has successfully achieved the bonding of many homogeneous or heterogeneous materials, such as Si-Si [17,18,19], SiC-SiC [20], Si-GaN [21] and Si-diamond [22]. Additionally, SAB has been used to bond GaN on some high thermal conductivity materials (such as SiC and diamond) for improving heat dissipation of GaN power devices [23,24], so it has extensive application prospects in the fields of advanced 5G communication, phased array radars, high-performance computing and new energy vehicles.

However, the investigation on the optimization of SAB process parameters is still very insufficient at present. Essig et al. [25] reported Si-GaAs direct bonding by SAB with high bonding energy of 900 mJ/m^2^ and a few interfacial voids but failed to reveal the effects of the process parameters on the bonding quality. There exists a similar situation in other studies related to SAB [13,21,23]. Overall, the previous studies usually only reported a certain process parameter scheme that could be used to realize SAB but did not clarify why such process parameters were selected. In our previous work [26], we studied the effect of surface activation time on Si-Si bonding quality and concluded that the activation time from 300 s to 420 s is the most suitable in terms of bonding strength and percentage of area covered by voids. In this work, the effects of Ar ion beam current on Si-Si bonding quality and thermal stability of bonding interface were systematically investigated, which had never been reported before.

## 2. Materials and Methods

N-type single-crystalline Si wafers (10 mm × 10 mm × 0.5 mm) with an extremely low root-mean-square (RMS) surface roughness of ~0.4 nm were chosen in the SAB experiment. Before Si-Si bonding, Si wafers were cleaned for 30 min by ultrasonic cleaning with acetone, absolute ethanol and deionized water in sequence. Then two cleaned wafers were mounted on the upper and lower specimen holders, respectively, in the bonding chamber of the SAB machine. After that, the chamber was vacuumed to 3.0 × 10^−5^ Pa. These two Si wafers were simultaneously irradiated for 300 s by an Ar ion beam with 260 V acceleration voltage and 45° incident angle, which could make the Si wafer surface highly reactive due to the generation of dangling bonds. Following the Ar ion beam irradiation, the surfaces of these two Si wafers were kept in contact with each other by the pressure of 0.8 MPa for 300 s, which resulted in the bonding of these two wafers. In order to investigate the effect of the Ar ion beam current on the Si-Si bonding quality, a series of beam currents were chosen in the irradiation step. The applied beam currents included 10 mA, 20 mA, 30 mA, 40 mA and 50 mA, whose corresponding ion fluxes were 1.2 × 10^19^ m^−2^ s^−1^, 2.4 × 10^19^ m^−2^ s^−1^, 3.6 × 10^19^ m^−2^ s^−1^, 4.8 × 10^19^ m^−2^ s^−1^ and 6.0 × 10^19^ m^−2^ s^−1^, respectively. In addition, the annealing treatment at 500 °C in Ar atmosphere for 10 min with a heating rate of 5 °C s^−1^ and a cooling rate of 2 °C s^−1^ was executed to evaluate the thermal stability of the bonding interfaces in the as-bonded Si-Si specimens.

The RMS surface roughnesses of the Si wafers without and with the surface activation treatment were measured by atomic force microscopy (AFM, Innova, Bruker, Billerica, MA, USA). A scanning acoustic microscope (SAM, D9500, Sonoscan, Elk Grove Village, IL, USA) and a universal testing machine (858 Mini Bionix. II, MTS, Eden Prairie, MN, USA) were used to estimate the percentages of area covered by voids in the bonding interfaces and the bonding strengths of the bonded Si-Si specimens, respectively. Transmission electron microscopy (TEM, JEM-F200, JEOL Ltd., Tokyo, Japan) and energy dispersive spectrometer (EDS, JEM-F200, JEOL Ltd., Tokyo, Japan) was adopted to analyze the nanostructures and chemical compositions of the Si-Si bonding interfaces, respectively.

## 3. Results and Discussion

### 3.1. Interfacial Void Analyses

**Figure 1:**
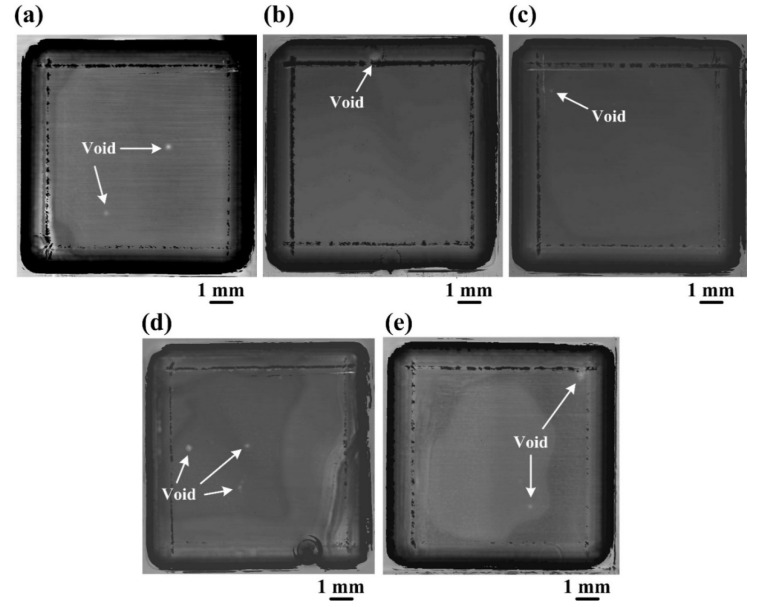
SAM images of the bonded Si-Si specimens activated under the Ar ion beam currents of (**a**) 10 mA, (**b**) 20 mA, (**c**) 30 mA, (**d**) 40 mA and (**e**) 50 mA, respectively.

**Figure 2:**
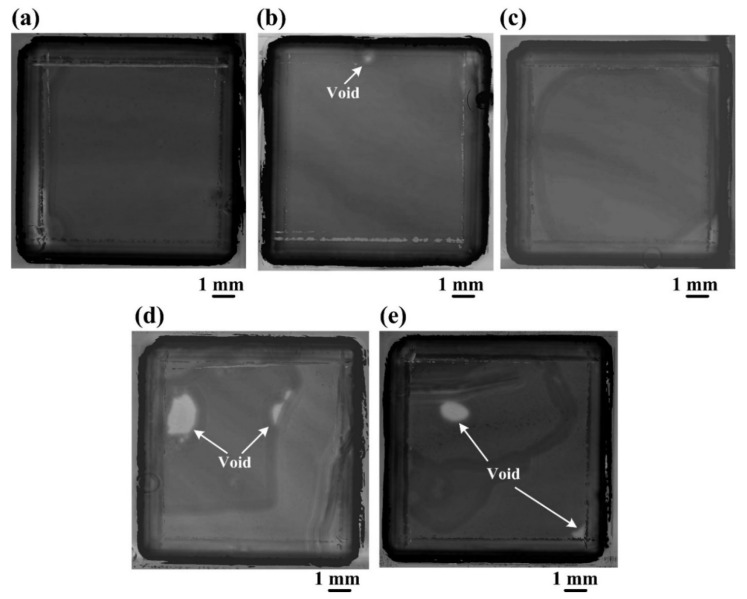
SAM images of the bonded Si-Si specimens activated under the Ar ion beam currents of (**a**) 10 mA, (**b**) 20 mA, (**c**) 30 mA, (**d**) 40 mA and (**e**) 50 mA, respectively, and having undergone the post-annealing treatment.

The bonded Si-Si specimens were successively tested for interfacial void analyses by SAM. The SAM images of the five bonded Si-Si specimens activated under different Ar ion beam currents before and after annealing treatment are shown in Figure 1 and Figure 2, respectively, and their percentages of the area covered by voids are listed in Table 1. All these specimens have only a few interfacial voids (1~3 voids) with a size of <0.5 mm and a low percentage of area covered by voids (≤0.5%). The three bonded Si-Si specimens activated under the Ar ion beam currents of 10 mA, 20 mA and 30 mA have lower percentages of the area covered by voids (<0.2%) in comparison with the other two specimens activated under 40 mA and 50 mA (~0.2% and ~0.5%). After the annealing treatment at 500 °C in the Ar atmosphere, the percentages of the area covered by voids of the specimens activated under the Ar ion beam currents from 10 mA to 30 mA decreased to zero or kept unchanged, while the other two specimens activated under 40 mA and 50 mA increased to ~3.5% and ~2%, respectively, as shown in Table 1. Therefore, a smaller Ar ion beam current from 10 mA to 30 mA for surface activation is beneficial to reduce the percentage of area covered by voids and improve the thermal stability of the Si-Si bonding interface.

For analyzing the reason for the result mentioned above, the surface roughnesses of an original Si wafer and five Si wafers activated under the Ar ion beam currents of 10 mA, 20 mA, 30 mA, 40 mA and 50 mA, respectively, were measured by AFM. However, all the five surface-activated Si wafers have the same RMS surface roughness of ~0.4 nm as the original Si wafer, which indicates the irradiation of the Ar ion beam in this current range has no effect on the surface morphology of the Si wafer, and the discrepancies between the percentages of the area covered by voids of these bonded Si-Si specimens are not caused by the differences of the surface roughnesses of the Si wafers. The lower percentage of area covered by voids and better thermal stability of the bonded Si-Si specimens activated under the ion beam current of 10~30 mA is attributed to the adequate and proper surface activation of Si wafers, which is described in Section 3.2.

### 3.2. Bonding Strength Testing

**Figure 3:**
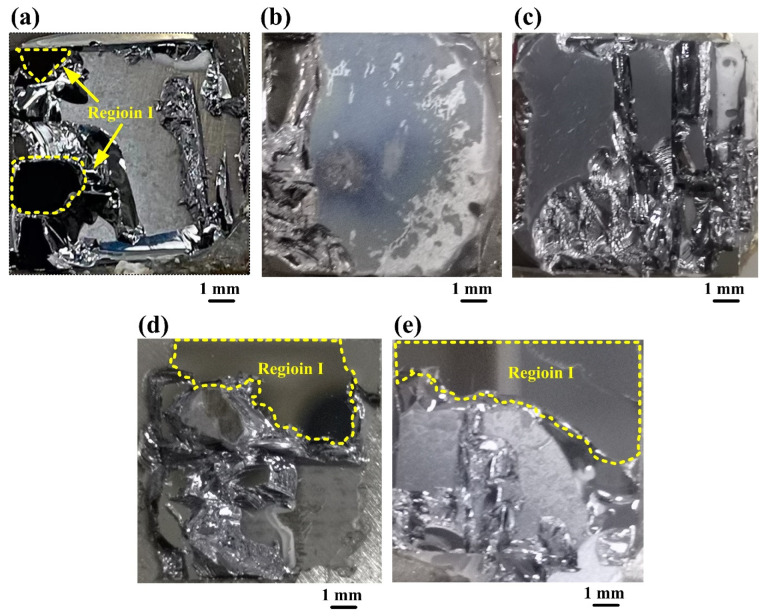
Surface profiles of the fractured parts from the bonded Si-Si specimens activated under the Ar ion beam currents of (**a**) 10 mA, (**b**) 20 mA, (**c**) 30 mA, (**d**) 40 mA and (**e**) 50 mA, respectively, after bonding strength testing.

**Figure 4:**
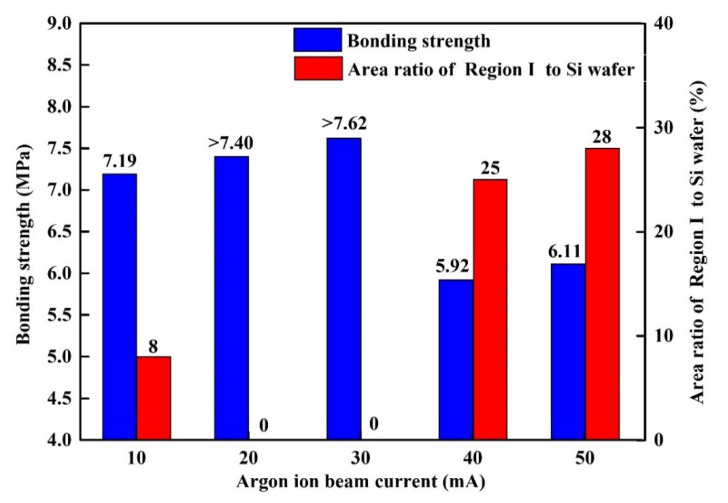
Bonding strengths and area ratios of Region I to Si wafer for the bonded Si-Si specimens.

A universal testing machine was used to evaluate the bonding strengths of the bonded Si-Si specimens. During the testing, the outer surfaces of the two wafers in a bonded Si-Si specimen were first adhered to the upper and lower specimen holders in this machine, respectively, with a high-adhesive-strength resin glue, and then these two holders were forced away from each other by hydraulic pressure with a speed of 0.5 mm s^−1^, which applied a tensile force perpendicular to the bonding interface until the specimen broke. The fracture strengths of the five bonded specimens are 7.19 MPa, 7.40 MPa, 7.62 MPa, 5.92 MPa and 6.11 MPa, respectively. Figure 3 shows the surface profiles of the fractured parts from the five bonded Si-Si specimens after the bonding strength testing. The regions I circled with the yellow dashed lines denote the regions fractured at the bonding interfaces of these specimens, while the other regions are the places fractured at solidified adhesive glue and bulk Si. For the specimens activated under the Ar ion beam currents of 20 mA and 30 mA, the regions fractured at the bonding interfaces could not be observed, which indicated the fracture occurred completely at the solidified adhesive glue and bulk Si. Therefore, the real bonding strength of each of these two specimens is both larger than their respective fracture strength. For this reason, the bonding strengths of the five bonded Si-Si specimens activated under the beam currents of 10 mA, 20 mA, 30 mA, 40 mA and 50 mA are 7.19 MPa, >7.40 MPa, >7.62 MPa, 5.92 MPa and 6.11 MPa, respectively, as shown with the blue columns in Figure 4. As can be seen, the bonding strength increases from 7.19 MPa to >7.62 MPa as the beam current rises from 10 mA to 30 mA but evidently decreases after over 30 mA. In terms of bonding strength, an Ar ion beam current between 10 mA and 30 mA during the surface activation is more suitable for Si-Si direct bonding.

As we know, Ar ion beam irradiation has two aspects of effects on Si wafer surface, including productions of dangling bonds and an amorphous Si layer on the surface of single-crystalline Si. When the beam current ranges from 10 mA to 30 mA, the number of dangling bonds generated by Ar ion beam irradiation is the main factor affecting the bonding strength. There are more doses of Ar ions bombarding the Si wafer surface during the irradiation of 300 s as the beam current increases from 10 mA to 30 mA so as to produce more dangling bonds, which can form more chemical bonds between the two activated Si surfaces contacting with each other and therefore enhance the bonding strength. However, the amorphous Si layer becomes thicker with the rise of the beam current, as demonstrated in Section 3.3, so that the effect of the amorphous Si layer on the bonding strength can not be neglected in the case of the beam current over 30 mA. In this situation, the thicker amorphous Si layer, instead of the number of dangling bonds, becomes the main factor affecting the bonding strength. The amorphous Si layer has a loose structure with high-density defects, so a thicker amorphous Si layer brings adverse impact to the bonding strength, which causes the Si-Si bonding strength to decrease when the Ar ion beam for surface activation exceeds 30 mA. Consequently, the surface activation under a relatively low Ar ion beam current from 10 mA to 30 mA is considered a better choice for achieving high Si-Si bonding strength.

The area ratio of Region I to Si wafer for each of these Si-Si specimens is shown in Figure 4 with a red column, basically exhibiting a variation tendency of first decreasing and then increasing with the Ar ion beam current rising from 10 mA to 50 mA. This area ratio is related to the bonding strength, which has a roughly opposite variation tendency for these Si-Si specimens. For a bonded Si-Si specimen, its higher bonding strength indicates a higher adhesion strength at its bonding interface, so its area ratio of Region I to Si wafer is lower. The area ratios of the two specimens activated under the Ar ion beam currents of 20 mA and 30 mA are both 0%, which means the interfacial adhesion strength of each of them is superior to that of bulk Si or solidified adhesive glue.

The maximum bonding strength (>7.62 MPa) of the Si-Si wafer direct bonding realized in this SAB experiment is superior to that (4.15 MPa) of the Si-Si wafer bonding via the wet chemical surface activation at 250 °C reported in the literature [27], which indicates the Si-Si wafers bonded with the SAB technique based on Ar ion beam irradiation can reach a relatively high bonding strength.

### 3.3. TEM Observation and EDS Analyses

**Figure 5:**
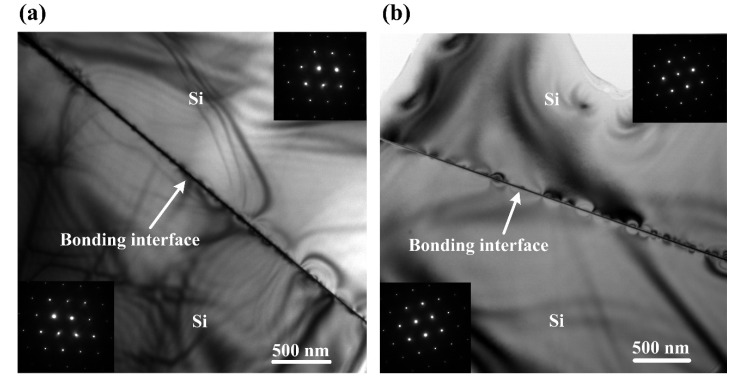
Low-magnification cross-sectional TEM images (10^4^ times) of the bonding interfaces with two illustrations showing the diffraction patterns of the Si wafers in the two bonded Si-Si specimens activated under the Ar ion beam currents of (**a**) 10 mA and (**b**) 30 mA.

TEM and EDS were used to characterize the bonding interfaces of the bonded Si-Si specimens. Figure 5a,b show the low magnification (10^4^ times) cross-section TEM images of the two bonded specimens activated under the Ar ion beam currents of 10 mA and 30 mA, respectively. As can be seen, there is no nanovoid at the two bonding interfaces.

**Figure 6:**
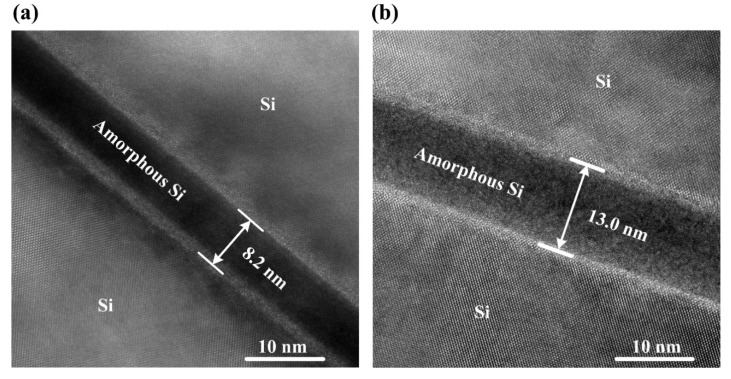
High-magnification cross-sectional TEM images (5 × 10^5^ times) of the bonding interfaces in the two bonded Si-Si specimens activated under the Ar ion beam currents of (**a**) 10 mA and (**b**) 30 mA.

In order to observe the details of the bonding interfaces, high-magnification (5 × 10^5^ times) cross-section TEM images were taken, as shown in Figure 6. It can be clearly seen that there exists an amorphous Si layer at both the bonding interfaces. The amorphous Si layer was generated by Ar ion beam irradiation during surface activation. Furthermore, the amorphous layer (approximately 13.0 nm) of the bonded Si-Si specimen activated under a beam current of 30 mA is obviously thicker than that (approximately 8.2 nm) of the one activated under 10 mA, which is attributed to the difference between the doses of Ar ions bombarding the Si wafer surfaces for these two bonded Si-Si specimens. A higher beam current means a larger dose of Ar ions bombarding the single-crystalline Si wafers, which makes these Si wafers suffer more serious surface lattice damage. Therefore, a larger Ar ion beam current for surface activation leads to a thicker amorphous Si layer at the bonding interface.

**Figure 7:**
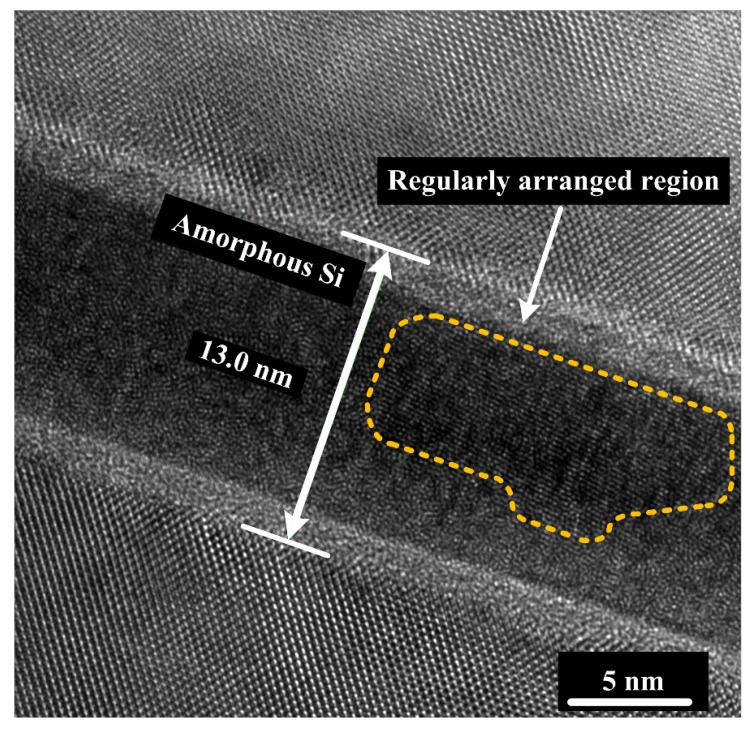
High-magnification cross-sectional TEM image (1.3 × 10^6^ times) of the bonding interface in the bonded Si-Si specimen activated under the Ar ion beam current of 30 mA.

**Figure 8:**
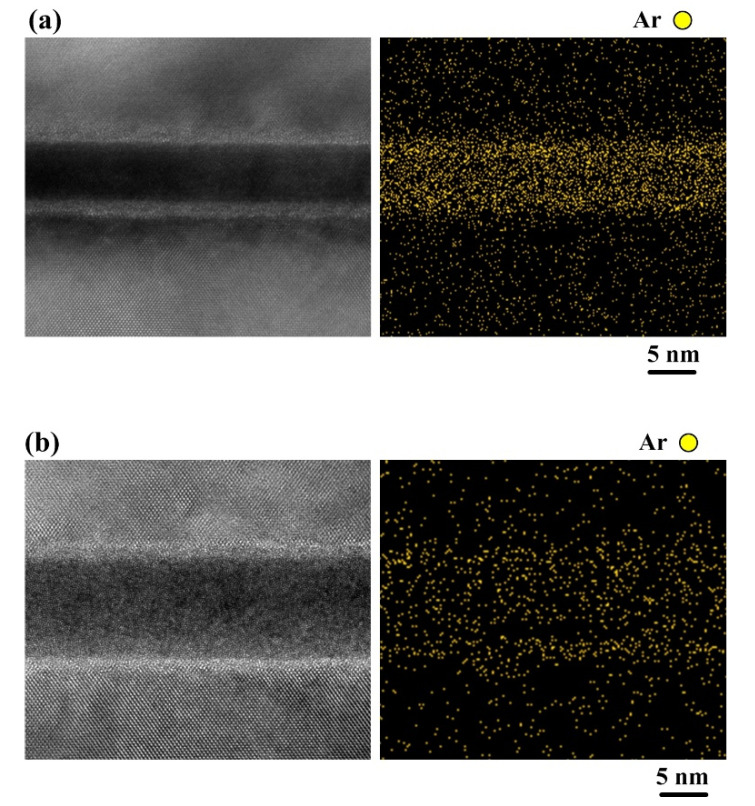
High-magnification cross-sectional TEM and high-resolution EDS mapping images of the bonding interfaces in the two bonded Si-Si specimens activated under the Ar ion beam currents of (**a**) 10 mA and (**b**) 30 mA.

Figure 7 is a 1.3 × 10^6^ times high magnification cross-section TEM image of the bonded Si-Si specimen activated under a beam current of 30 mA. Most region of the bonding interface is in an amorphous state, but there exists a special region with Si atoms arranged regularly circled with a yellow dashed line, whose formation cause and effect on the Si-Si bonding quality are still under investigation. In addition, Figure 8a,b show the high-resolution cross-section EDS mapping images of the two bonded Si-Si specimens activated under the Ar ion beam currents of 10 mA and 30 mA, respectively. Ar element is distributed in the amorphous Si layer due to Ar ion injection during the Ar ion beam irradiation.

## 4. Conclusions

The effect of argon ion beam current for surface activation on the Si-Si bonding quality was investigated. The bonded Si-Si specimens activated under a lower beam current from 10 mA to 30 mA have a lower percentage of area covered by voids, better thermal stability, and higher bonding strength, so this beam current is more suitable for Si-Si surface activated bonding. For the bonded Si-Si specimen activated under a beam current of 30 mA, its percentage of area covered by voids and bonding strength reach <0.2% and >7.62 MPa, respectively. TEM results indicate that there is an amorphous Si layer at the bonding interface created by Ar ion beam irradiation, and it becomes thicker as the beam current becomes larger. These experimental results are expected to be helpful for the optimization of the SAB process.

## Figures and Tables

**Table 1:** Percentages of area covered by voids of the bonded Si-Si specimens before and after annealing treatment.

Ar Ion Beam Current (mA)	Percentage of Area Covered by Voids (%)	
	Before Annealing	After Annealing
10	<0.2	0
20	<0.2	<0.2
30	<0.2	0
40	~0.2	~3.5
50	~0.5	~2

## Data Availability

Data are contained within the article.
